# Clinical spectrum and long-term follow-up of 14 cases with *G6PC3* mutations from the French severe congenital neutropenia registry

**DOI:** 10.1186/s13023-014-0183-8

**Published:** 2014-12-10

**Authors:** Claire Desplantes, Marie Louise Fremond, Blandine Beaupain, Jean Luc Harousseau, Agnès Buzyn, Isabelle Pellier, Gaelle Roques, Pierre Morville, Catherine Paillard, Julie Bruneau, Lucile Pinson, Eric Jeziorski, Jean Pierre Vannier, Capucine Picard, Florence Bellanger, Norma Romero, Loïc de Pontual, Hélène Lapillonne, Patrick Lutz, Christine Bellanné Chantelot, Jean Donadieu

**Affiliations:** Service de Pédiatrie CHU de Strasbourg, Strasbourg, France; Service de Pédiatrie, APHP Hôpital Jean Verdier, Bondy, France; AP-HP, Registre français des neutropénies chroniques sévères, Centre de référence des déficits Immunitaires Héréditaires, Service d’Hémato-oncologie Pédiatrique Hôpital Trousseau, Paris, France; Service d’Hématologie, CHU de Nantes, Nantes, France; AP-HP Service d’hématologie Hôpital Saint Antoine, Paris, France; Service de Pédiatrie Hémato immunologie CHU Angers, Angers, France; Service d’Onco-Hématologie Pédiatrique, Hôpital des enfants, CHU de Reims, Reims, France; Service de Cardiologie pédiatrique, Hôpital des enfants, CHU de Reims, Reims, France; Laboratoire d’Anatomie et Cytologie Pathologique, APHP Hôpital Necker Enfants Malades, Paris, France; Service de génétique, Hôpital Arnaud de Villeneuve, CHU Montpellier, Montpellier, France; Service de Pédiatrie Hôpital Arnaud de Villeneuve, CHU Montpellier, Montpellier, France; Service de Pédiatrie Hémato Oncologie, CHU Rouen, Rouen, France; Centre d’Etude des Déficits Immunitaires, APHP, Hôpital Necker-Enfants Malades, Paris, France; AP-HP, Hôpital Pitié-Salpêtrière, Département de Génétique, Université Pierre et Marie Curie, Paris, France; Unité de Morphologie Neuromusculaire Institut de Myologie Inserm UMRS 974 - CNRS UMR 7215 UPMC Paris 6, GHU Pitié-Salpêtrière 47, boulevard de l’Hôpital, F-75 651 Paris Cedex 13, France; AP-HP, laboratoire d’hématologie, Hôpital Trousseau, UMPC Univ Paris 06 and INSERM UMR_S938, proliferation and differentiation of stem cells, F-75012 Paris, France

## Abstract

**Background:**

The purpose of this study was to describe the natural history of severe congenital neutropenia (SCN) in 14 patients with *G6PC3* mutations and enrolled in the French SCN registry.

**Methods:**

Among 605 patients included in the French SCN registry, we identified 8 pedigrees that included 14 patients with autosomal recessive *G6PC3* mutations.

**Results:**

Median age at the last visit was 22.4 years. All patients had developed various comordibities, including prominent veins (n = 12), cardiac malformations (n = 12), intellectual disability (n = 7), and myopathic syndrome with recurrent painful cramps (n = 1). Three patients developed Crohn’s disease, and five had chronic diarrhea with steatorrhea. Neutropenia was profound (<0.5 × 10^9^/l) in almost all cases at diagnosis and could marginally fluctuate. The bone marrow smears exhibited mild late-stage granulopoeitic defects. One patient developed myelodysplasia followed by acute myelogenous leukemia with translocation (18, 21) at age 14 years, cured by chemotherapy and hematopoietic stem cell transplantation. Four deaths occurred, including one from sepsis at age 5, one from pulmonary late-stage insufficiency at age 19, and two from sudden death, both at age 30 years. A new homozygous mutation (c.249G > A /p.Trp83*) was detected in one pedigree.

**Conclusions:**

Severe congenital neutropenia with autosomal recessive *G6PC3* mutations is associated with considerable clinical heterogeneity. This series includes the first described case of malignancy in this neutropenia.

## Introduction

Severe congenital neutropenia (SCN) encompasses a group of rare diseases associated with neutropenia and other developmental defects. To date in 2014, a total of 18 genes have been identified as responsible for this entity [1]. In 2009, Botzug et al. [2] described a subgroup of SCN patients with biallelic *G6PC3* mutations, encoding the catalytic subunit 3 of glucose-6-phosphatase. In addition to severe neutropenia, this group of patients exhibits three major features: skin abnormalities with a visible perivenous system, cardiac abnormalities (mainly atrial septal defect), and urogenital abnormalities. By 2014, this entity, also termed SCN 4 or Dursun syndrome, had been reported in 61 cases [3-7]. Based on enrollment in the French SCN registry, we identified 14 cases from eight pedigrees, including two pedigrees previously reported prior to gene identification. Indeed, in 1994, Stoll *et al*. [8] described a new syndrome associating facial dysmorphia, skin disorder, cardiac and/or urogenital abnormalities, recurrent infections, and neutropenia. Previously, in 1982, Vannier *et al*. reported a case associating Crohn’s disease and neutropenia [9], the first such case reported. This series describes the long-term outcome of such patients and describes several adverse consequences such as leukemia and death and also one pregnancy, expanding the phenotypic picture of this disease.

## Patients and methods

### Organization of the French registry and data monitoring

All patients included in this study were registered in the French Severe Chronic Neutropenia Registry, created in 1993 and based on prospective enrollment. We included all types of congenital neutropenia [1]. The registry received national certification in 2008 by the French health authorities, and 35 French regional pediatric hematology–oncology clinical units participate in it. Data monitoring was based on a review of medical records collected by a clinical research associate who visited each center yearly. The patient or a legal guardian provided written informed consent before being included in the registry. Several reports of the registry are available elsewhere [10,11]. In addition to information about the hematological outcome, we collect information on patient morphological features. *G6PC3* mutations were suspected if a patient presented with a congenital neutropenia associated with at least one additional non-hematologic aberration, such as increased superficial venous markings, congenital heart disease, or urogenital anomalies. Patient UPN5248 has already been described as patient #9 in the initial description [2]; patients UPN5192, UPN5193, and UPN5194 were described in 1994 [8]; and patient #4 was included in a survey about hematopoietic stem cell transplantation in congenital neutropenia [12]. Finally, patient UPN5273 was patient #1 in the first report describing this syndrome worldwide in 1982 [9].

### Clinical investigation

Demographic, auxologic, nutritional, and hematological information was collected, as were results of liver tests and immunological tests, and infectious history. Septicemia, cellulitis, bacterial or fungal pneumonia, osteitis, pyelonephritis, and liver abscess were considered as severe infections and systematically recorded, as were causative germs. Minor infections were those for which patients did not seek medical surveillance such as stomatological or ear, nose, and throat infections. These events were often omitted in the medical records. Immunoglobulin (Ig) levels were analyzed according to age. Age at diagnosis was defined by the age at the first pathological manifestation leading to the diagnosis of chronic neutropenia.

### Definition of hematological features and hematological complications

The initial complete blood count (CBC) value was the median value of the three first CBCs collected in each patient’s lifetime. Baseline CBC was considered if samples were collected during routine consultations, with the exception of periods involving granulocyte colony-stimulating factor (G-CSF) therapy and any hematological complications as defined below. The World Health Organization 2008 classification was used to define acute leukemia. Myeloid blockage was defined according to a previous study [13].

### Clinical phenotype

We routinely analyzed parameters such as auxologic and major medical events requiring medical management. We collected clinical information on facial features, skin aspect, heart and urogenital anomalies, and any other malformations.

### *G6PC3* gene sequencing

The patients or their parents gave written informed consent for genetic testing. Genomic DNA was extracted from blood with standard procedures. The coding sequence and exon–intron boundaries of the *G6PC3* gene were amplified using primers and PCR conditions described previously [2]. Purified PCR products were sequenced in both directions using the BigDye Terminator chemistry (Life Technologies, Saint-Aubin, France) on an ABI3730 Genetic Analyser. Sequences were analyzed with the Seqscape software v2.2. We numbered mutations as recommended by the Human Genome Variation Society (http://www.hgvs.org/), using the reference sequence NM_003467.2. Among the 605 patients with SCN included in the registry, *G6PC3* mutations were screened in 85 unrelated patients. G6PC3 was screened if neutropenia was associated with comorbidities like a congenital heart defect or prominent veins and if *ELANE* and *SBDS* mutations had been previously excluded.

### Statistical analysis

Stata software version 10 was used for all statistical analyses. Lower and upper interquartile (p25 and p75, respectively) and median values were used to depict the distribution of quantitative variables. For survival analysis, the endpoint was death. The period taken into account was the time interval from birth to the first date when the event was observed or to the last examination when no event occurred. The Kaplan–Meier method was used to estimate survival rates, with a cut-off date of April 1, 2014.

### Literature review

To identify all publications related to G6PC3-associated neutropenia to summarize reported cases, we first screened PubMed, with the key words ‘G6PC3 and neutropenia or Dursun syndrome’. We then checked the bibliography of each article to identify additional references and avoid duplicates.

## Results

### Demographic data

Fourteen patients including eight males, originating from eight families, were identified among 605 patients (2.1%) included in the French SCN registry. The median age at last follow-up was 22.4 years [min = 4; max = 50]. The total follow-up period was 308 patient years.

### Genetics

**Figure 1:**
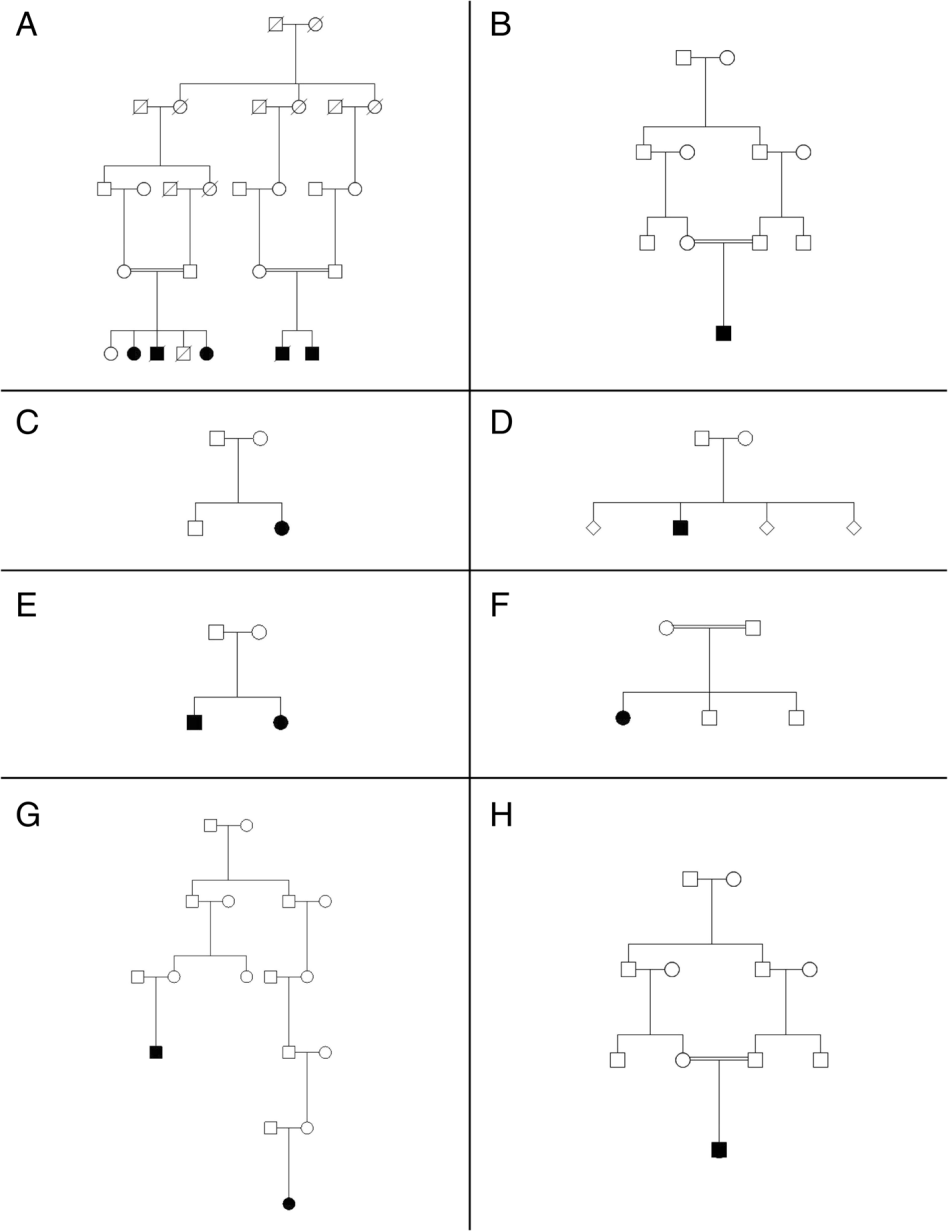
**Genealogic tree of the eight described families.** Filled-in circles or squares represent affected cases with *G6PC3* neutropenia. Each pedigree are designed by a letter (**A** to **H**).

We analyzed eight families, six originated from France and two from North Africa (Algeria). Pedigree study demonstrated common ancestors in two families (Figure 1A, F) where cases were found in apparently separated branches. We identified a homozygous *G6PC3* mutation in seven families. Four of them were truncating mutations (W83* (family A), R161* (D), and R189* in pedigrees G and H), and two were missense mutations (R253H and G260R in families B, E, and F). We identified a compound heterozygote genotype ([c.[829C > T];[677 + 1G > A], p.[Q277*]; [?]) in pedigree C (Figure 1, Table 1). This proband (pedigree C, Table 1) has been previously described (#9) [2]. All identified *G6PC3* mutations also have been previously described except the nonsense mutation W83* in exon 2.

**Table 1:** **Summary of the clinical and biological features of the 14 patients**

**Pedigree**	**UPN**	**Sex/age/vital status**	**G6PC3 genotype**	**Age at diagnostic**	**Median ANC/AMC**	**Others hematological abnormalities**	**Therapy**	**Severe infections**	**Cardiac abnormalities**	**Urogenital abnormalities**	**Cutaneous abnormalities**	**Digestive tract**	**Other findings**	**Neurodev. difficulties**
A	5194	F/36/L	c.[249G > A];[249G > A] p.[W83*];[W83*]	New born	ANC: 0.7	Mild Thrombocytopenia/Mild anemia prior leukemia/AML at age of 14 y	HSCT	Yes	Aortic insufficiency	Grade III RVU	Prominent veins, cutis laxa	Steatorrhea	Heart failure	Yes
					AMC: 0.28					urethral duplication			Frontal bossing, thick lips, prognathism Respiratory failure Hypothyroidism	
	5193	M/17/D		New Born	ANC 0.383		G-CSF	Yes Repeated pneumonitis with bronchiectasis and lethal respiratory insufficiency	ASD/surgery	Bilateral cryptorchidism	Prominent veins, cutis laxa	Steatorrhea	Frontal bossing, thick lips, prognathism	Yes
					AMC: 1.44					Hypospadias				
	5192	F/29/L		New Born	ANC 0.411	Mild Anemia Mild Thrombocytopenia	G-CSF	Yes	ASD/surgery	Bilateral grade I RVU	Prominent veins, cutis laxa	Steatorrhea	Respiratory failure	Yes
					AMC 1.11								thick lips, prognathism	
	5131	M/30/D		New Born	ANC: 1.368 AMC 0.55	Mild Anemia	No	No	PDA Overriding aorta	Grade III RVU right side Cryptorchidism	Prominent veins, cutis laxa	IBD Steatorrhea	Thick lips, prognathism Pulmonary arterial hypertension Polyarthritis	Yes
									Death/sudden death					
	5130	M/24/L		New born	ANC 0.314	Mild Anemia	G-CSF	Yes	WPW Sd	Cryptorchidism	Prominent veins,cutis laxa	Steatorrhea	Bilateral hearing loss	Yes
					AMC 0.92					Bilateral RVU			Congenital right ptosis, prominent lips, abnormal ear	
										Megaureter				
B	6262	M/13/L	c.[758G > A];[758G > A];	7 months	ANC 0.54	Mild anemia	G-CSF	Yes stomatitis Pneunonitis	No	No	Prominent veins	m	Kabuki syndrome like	Yes
			p.[R253H];[R253H]		AMC 0.56								Portal cavernoma	
													Cerebral palsy	
C	5248	F/19/L	c.[829C > T];[677 +1G > A]	New born	ANC 0.3	No	G-CSF	Yes	Mild dilatation of ascendant aorta	No	Prominent veins	No	Myopathy Polyarthritis	No
			p.[Q277*]; [?]		AMC 1.276								Raynaud Hyperopia Gastroesophageal reflux urinary incontinence, Loose stools	
D	5643	M/17/L	c.[481C > T];[481C > T]	New born	ANC 0.405	Mild Anemia	G-CSF	Yes	Aortic insufficiency	Cryptorchidism micropenis	Prominent veins	Failure to thrive	Narrowed thorax	No
			p.[R161*];[R161*]		AMC 0.31	Mild Thrombocytopenia	LD steroid					Steatorrhea	Inguinal hernia	
												IBD		
E	6536	M/16/L	c.[778G > C];[778G > C]	New born	ANC 1.071		No	Yes	ASD Aortic insufficiency	Cryptorchidism	Prominent veins	No	Frontal bossing, board nasal bridge, jug ear Right strabism Umbilical hernia	No
			p.[G260R];[G260R]		AMC: 0.41				Tricuspid regurgitation/surgery	Bilateral RVU			Bilateral deafness	
	6420	F/3/L		New born	ANC 0.4		No	No Yes but no with antibiotic prophylaxis	ASD Primary pulmonary hypertension	No	No	No	Board nasal bridge	No
					AMC 0.756									
F	5273	F/50 L	c.[778G > C];[778G > C]	7 months	ANC 0.7	Severe Anemia	G-CSF	Yes Pneumonitis cellulitis	ASD/surgery	Cryptorchidism	Prominent veins	IBD	HTAP, dwarfism, Deafness	No
			p.[G260R];[G260R]		AMC 0.5		LD steroid			Bilateral RVU				
G	5340	F/29/L	c.[565C > T];[565C > T]	New born	ANC 0.52	No	G-CSF	Yes	ASD/surgery	A normal pregnancy with normal child	Prominent veins	No	Very high voice	No
			p.[R189*];[R189*]		AMC 0.34									
	5847	M/30/D		4.5 years	ANC 0.565	Severe Thrombocytopenia	No	Yes	ASD/surgery planned	Cryptorchidism	Prominent veins	No	Precocious pubic hair growth and delayed puberty	No
					AMC 0.16	Mild Anemia			Death/sudden death after sport activity no autopsy	micropenis				
H	5805	M/5 D	c.[565C > T];[565C > T]	New born	ANC 1.03	Mild Anemia	GCSF	Yes sepsis	No	No	Prominent veins	Failure to thrive Enteral nutriti on gastrostomy	Pierre robin sequence	Major intellectual disability with bilateral sus-tentorial atrophy on MRI
			p.[R189*]; [R189*]		AMC 0.69	Mild Thrombopenia		Lethal pyocyanic sepsis					Brachiocephalic thrombosis	

### Features at birth and clinical presentation

The median age at first manifestation was 0.02 years. Initial diagnoses were very heterogeneous—Kabuki syndrome in one case, Pierre Robin sequence suspected in one but not confirmed, Shwachman-Diamond syndrome in three, and Fanconi anemia in two—while in the other cases, the disease remained unclassified for years. The median term at birth was 37.5 weeks (interquartile range (IQR): 36–40 weeks), and five babies (35%) were premature, being born between 30 and 35 weeks of gestational age. The median birth weight was 2390 g (IQR: 2155–2620), median length was 46 cm (IQR: 44–48), and median head circumference was 32 cm (IQR: 31.5–35). Six (42%) babies were considered to have had intrauterine growth retardation.

### Hematologic and immunological features

At the time of diagnosis, the median white blood cell count (WBC) was 5.3 × 10^9^/l (IQR 2.5–7.2 × 10^9^/l), absolute neutrophil count (ANC) was 0.59 × 10^9^/l (IQR: 0.2–1.97 × 10^9^/l), absolute lymphocyte count (ALC) was 2.7 × 10^9^/l (IQR: 1.7–4.3 × 10^9^/l), and absolute monocyte count (AMC) was 0.7 × 10^9^/L (IQR 0.37–2.05 × 10^9^/l). On the first available CBC, ANC was below 0.05 × 10^9^/l in seven patients, between 0.5 and 1 × 10^9^/l in three patients, and four patients had a count above 1.5 × 10^9^/l. The median hemoglobin (Hb) level was 13.8 g/dl (IQR: 11.7–18.9 g/dl), and platelet count was 177 × 10^9^/l (IQR: 173–297 × 10^9^/l). During routine follow-up, the ANC fluctuated with time, without any detectable regular variation. Taking into account all the available CBCs recorded during the routine follow-up of the patients (with the exception of the G-CSF therapy period), with a median number of 23 per patient, the median WBC was 4 × 10^9^/l (IQR: 2.7–4.5 × 10^9^/l), ANC was 0.53 × 10^9^/l (IQR: 0.4–0.7 × 10^9^/l), ALC was 1.7 × 10^9^/l (IQR: 1.6–2.4 × 10^9^/l), and AMC was 0.55 × 10^9^/l (IQR: 0.34–0.9 × 10^9^/l). The median Hb level was 12 g/dl (IQR: 10.9–13 g/dl), and the median platelet count was 280 × 10^9^/l (IQR: 239–353 × 10^9^/l). All patients had presented at least one CBC with a neutropenia below 0.5 × 10^9^/l during the follow-up.

**Figure 2:**
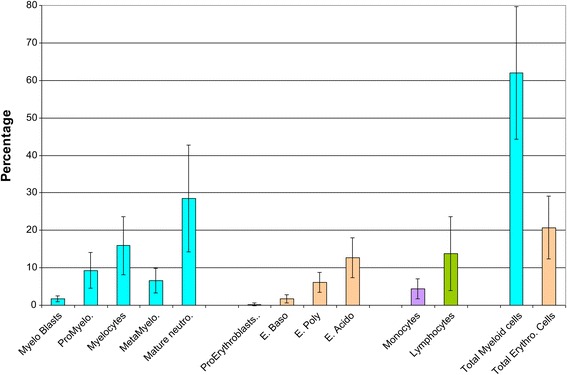
**Bone marrow mean value of the differential count with standard deviation (n = 13).**

**Figure 3:**
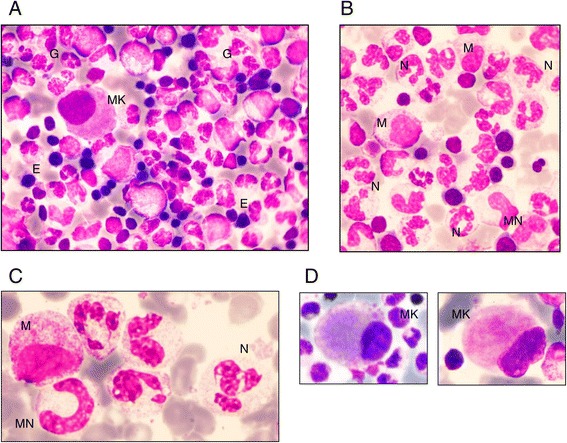
**Bone marrow morphology in patients with G6PC3 congenital neutropenia. (A)** Typical global bone marrow morphology: rich cellularity with predominant granulopoiesis (G), some erythroblasts (E), and one micromegakaryocyte (MK). **(B)** Predominant granulopoiesis with some myelocytes (M), very few metamyelocytes (MN), and a high number of mature neutrophils (N). **(C)** Details of neutrophils: hypersegmented appearance with thin opening between lobes and chromatin clumps. **(D)** Examples of micromegakaryocytes.

Bone marrow smears, evaluated in 13 patients, were centrally reviewed (HL) and globally failed to demonstrate a bone marrow blockage (Figure 2). In almost all cases, bone marrow was rich. The median percentage of myeloid lineage was 66%, which was quite high compared to the normal range (44–50%) and could correspond to a prolonged retention of neutrophils in the bone marrow. We observed a particular aspect of maturation impairment compared to the usual aspect in congenital neutropenia. Despite a normal percentage of mature neutrophils, there was a relatively low rate of metamyelocytes during the myeloid differentiation. In most cases (8/10), the morphologic aspect of myeloid neutrophils was unusual too, with nuclear hypolobulation or a hypersegmentation with thin opening between lobes, while the chromatin appeared condensed in separated clumps. There was no significant vacuolization of myeloid cells. This particular cell morphology can be considered to reflect myelokathexis. Megakaryocytic differentiation showed dysplastic manifestations with 10% being micromegakaryocytes. The erythroid lineage was normal in most cases, except for a macrocytosis tendency. Therefore, the most characteristic morphological features were presence of micromegakaryocytes and of neutrophils with hyperdense chromatin clumps, and a usually normal percentage of myeloid cells with an apparent lack of metamyelocytes (Figure 3).

Two patients developed severe transient anemia below 7 g/dl, and nine presented with mild transient anemia between 7 and 10 g/dl. Thrombocytopenia was commonly observed in our series, as nine developed at least one episode of thrombocytopenia (severe <50 × 10^9^/l in three, mild between 50 and 150 × 10^9^/l in six).

Plasma IgG was high in all patients (>3 SD) with a median level (age >5 years) of 15.75 g/l (IQR: 13–18) while median IgA was 0.7 g/l and median IgM was 1.07 g/l, in the normal range. Because of chronic bronchiectasis in one patient lasting for 6 years, intravenous Ig was provided despite a high IgG level, without any effect on the lung destruction; ultimately, the lung disease was the cause of death. Crohn’s disease (n = 3) and chronic arthritis (n = 1) were observed, but no other autoimmune manifestations were identified, and when assessed in three cases, no autoantibodies were detected.

The lymphocyte phenotypes were roughly normal in distribution. Counts were normal in six but low in four evaluable patients (Table 2). However, the distribution of naive T cells was diminished for the two patients in whom this was tested. This low proportion of naive T cells was present even in the absence of Crohn’s disease and regardless of age and immunosuppressive therapy.

**Table 2:** **Immunophenotype of 4 patients with** ***G6PC3*** **severe chronic neutropenia**

**UPN Age**	**527 3 age 50**	Range of normal values for an adult*	**5805 age 3**	Range of normal values at age 3*	**6536 age 1**	Range of normal values at age 1*	**6262 age 6**	Range of normal values for at age 6*
IgG/A/M in g/dL	9.7	7.68-16.3	15.6	4.2-10.9	4.35.	3.32-11.6	18.7	6.08-12.29
	1.7	0.68-3.78	0.55	0.22-1.57	0.32	0.14-1.05	1.7	0.33-2
	1	0.60-2.63	0.7	0.45-2.63	0.34	0.45-1.90	1.7	0.46-1.97
Antibodies against vaccine antigen	++		++				++	
Lymphocytes	700	1400–3300	831	2300–5400	3100	3600–8900	2800	2300–5400
CD3+	378	1200–2000	698	1400–3700	1891	2100–6200	1960	2100–6200
CD4+	252	530–1300	274.23	700–2200	1178	1300–3400	812	1300–3400
CD8+	98	330–920	332.4	490–1300	682	620–2000	756	490–1300
CD19+	49	110–570	58.17	390–1400	651	720–2600	364	390–1400
CD16+ CD56+	378	70–480	698.04	130–720	1891	180–920	1960	130–720
	Distribution of naive/memory T cells							
CD31+ CD45 + CD4+ (N 43–55%)	2						25	
CD45+CD4+ (N 43–55%)	19		37		27		50	
CCR7 + CD45RA+/CD8+ (N 52–63%)	5						43	
CCR7 +CD45RA-/CD8+ (N 3–14%)	7						6	
CCR7 -CD45RA-/CD8+ (N 20–41%)	78						35	
CCR7 -CD45RA+/CD8+ (N 11–26%)	10						16	

*in gr/dl for immunoglobulins and in μL×10^-3^ for lymphocytes.

### Infection

Of 14 patients, 11 presented with at least one episode of severe bacterial infection. A total of 64 episodes were registered for the 11 patients. We observed no severe viral infection. Various types of severe bacterial infections were observed: cellulitis (n = 22), pneumonitis (n = 22), osteitis (n = 2), mastoiditis (n = 3), pyelonephritis (n = 10), colitis with peritonitis (n = 1), and septicemia (n = 4). The median age at the first severe infection was 13.1 years with a large range from birth to age 29.7 years. Microbes were documented in 31 episodes, and the most common were Gram-negative bacteria in 17 episodes (*E. coli*, n = 7; *P. aeruginosa*, n = 5; *K. pneumoniae*, n = 3; and other Gram-negative species = 2) and Gram-positive cocci in 12 episodes (*S. aureus*, n = 6; pneumonia, n = 2; others, n = 4); no *Aspergillus* spp. were identified, and candidiasis was identified in two episodes.

In addition, all patients experienced multiple mild infections early in childhood, mainly otitis, pharyngitis, bronchitis, and gastroenteritis. Stomatitis was reported in eight patients. One patient died during a sepsis episode involving *Pseudomonas aeruginosa*. For two patients, bronchiectasis complicated the pulmonary infection, and these patients suffered from pulmonary insufficiency. One of these patients died from acute respiratory failure at age 17 years.

### G-CSF therapy and antibiotic prophylaxis

Antibiotic prophylaxis was common in the patients, based on long-term use of cotrimoxazole (n = 9) or alternate combined oral therapy (n = 2) but had a limited use for preventing severe infections, neither bronchiectasis or inflammatory bowel disease (IBD).

Nine patients were treated with G-CSF at a median dose of 6.8 μg/kg and a cumulative dose of 867 μg/kg of G-CSF per patient. In one patient, the response was poor to G-CSF at a dose of 28 μg/kg/day while the response was considered satisfactory in the other eight patients, with a median neutrophil count varying from 0.533 X 10^9^/l before G-CSF to 1.433 × 10^9^/l with G-CSF (*P* < 0.001). G-CSF always was indicated for infection prevention. In case of Crohn’s disease or IBDs, it appears to not have been successful in limiting symptoms such as pain and chronic diarrhea. Of note, oral steroids were prescribed to three patients for arthritis and IBD, resulting in a constant increase in neutrophils.

### Leukemia

One case of leukemia was observed. Patient UPN5194 was diagnosed at birth with aseptic meningitis. Neutropenia was diagnosed as well as an aortic valvular insufficiency. She later developed recurrent infections, including bronchitis, otitis, gastroenteritis, pharyngitis, osteitis, and urinary infections. When she was one year old, a bilateral vesico-ureteral reflux with bifid ureter was diagnosed. Mild developmental delay was first noted in the first year of life and improved later. When she was 13 years old, blast cells were detected on blood examination performed for pneumonitis. The bone marrow smear showed 13% myeloblasts with dysgranulopoiesis. Acute myeloblastic leukemia (AML) type M2 (FAB classification) was diagnosed 3 months later. The bone marrow karyotype revealed a t(8;21)(q22;q22) translocation. Bone marrow transplantation was performed when the patient was age 14 years using bone marrow from a healthy HLA-identical sister and a conditioning regimen with cyclophosphamide and busulfan. A relapse of the AML was observed 12 months later, treated by two courses of an AML-like chemotherapy regimen, and a second transplant was performed with the same donor and total body irradiation and a cyclophosphamide regimen. No graft-versus-host disease (GVHD) or relapse was observed, and the neutrophil count recovered after the transplant with full engraftment. The long-term outcome showed a full donor engraftment without GVHD, but chronic cardiomyopathy was observed while the skin appearance and dysmorphia remained. The patient was not able to become pregnant, and this hypofertility could have been related to the conditioning regimen.

### Comorbidities

All patients in our survey had one or several comorbidities, as shown in Table 1.

### Cardiac abnormalities

Cardiac abnormalities were observed in 12 of our patients: an atrial septal defect in seven patients, another abnormality in four (aortic insufficiency in three and mild dilatation of the ascending aorta in one), and a congenital rhythm abnormality (Wolff-Parkinson-White) in one. Pulmonary hypertension was present in three patients. The heart defect was always severe, requiring surgery in five cases. In addition to these presentations, two patients died suddenly in the third decade. In these cases, no autopsy was performed, and no formal causes of death can be defined; however, the circumstances of the deaths were the same. After physical activity during the day, death occurred during sleep (at age 30 years for both), which suggests a paroxysmal heart rhythm abnormality.

### Myopathy

**Figure 4:**
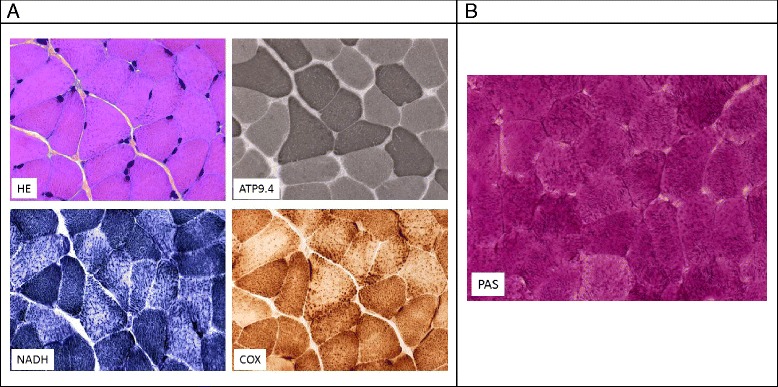
**Pathological features of the muscle in patient UPN5248 with myopathic syndrome. (A)** Serial skeletal muscle sections of biopsy from patient 1 showed mild variation in fiber size (HE = hematoxylin and eosin stain) and type 1 muscle fiber predominance (ATPase 9.40). With oxidative enzyme reactions (NADH, COX), the mesh of the intermyofibrillar network appeared thickened and slightly clumped, especially in type 2 fibers, which exhibit mainly glycolytic metabolism. **(B)** Skeletal muscle section of a biopsy from patient stained with PAS stain, showing intense coloration in all muscle fibers.

A myopathic syndrome was observed in one female (UPN5248/pedigree C). She was born at 39 weeks of gestational age, weighing 2380 g (<3 SD) and at 44 cm in length. Neutropenia was observed from age 7 days (ANC 0.3 × 10^9^/l). Her medical history was that of chronic severe neutropenia, with venous skin visibly obvious from age 3 years. Despite normal psychomotor development, sitting was acquired at 15 months and walking at age 20 months. At age 2.5 years, she developed recurrent episodes of muscle pains and cramps localized to the proximal muscles, particularly the quadriceps. Such cramps were intermittently observed with a number of episodes from one per week to one per month and were still present at the last update when she was age 20 years. Initially, the cramps were considered to be related to G-CSF therapy but appeared later to be independent of it. Muscle strength was normal, but such episodes could be aggravated by sport activity or even with limited muscle effort. Because the etiology was not determined from age 14 years, the association of the muscle cramps and neutropenia has been extensively explored. None of the metabolic studies, including complete sequencing of the mitochondrial DNA, showed abnormalities. Several muscle biopsies were performed. Skeletal muscle sections of biopsy showed mild variation in the fiber size (hematoxylin and eosin stain) and type 1 muscle fiber predominance (ATPase 9.40). With oxidative enzyme reactions for nicotinamide adenine dinucleotide (NAD COX), the mesh of the intermyofibrillar network appeared thickened and slightly clumped, especially in type 2 fibers, which exhibit mainly glycolytic metabolism. PAS staining showed intense coloration in all muscle fibers, suggesting glycogen accumulation (Figure 4).

### Urogenital abnormalities

Five patients required surgery because of severe vesico-ureteral reflux. Six male patients had cryptorchidism. Other urogenital anomalies were epispadias (n = 1), hypospadias (n = 1), and megaureter (n = 1).

### Cutaneous abnormalities

Increased visibility of superficial veins is a known clinical feature in G6PC3 deficiency, and 13 of our patients presented with this disorder. In pedigree E, consisting of two patients, only one had a prominent superficial venous pattern. *Cutis laxa* was noted in all patients of family A. In two patients from this pedigree, skin biopsy, unfortunately not available for a second review, showed a disappearance of the hypodermis with collagen fiber hypertrophy.

### Nutrition and digestive tract

**Figure 5:**
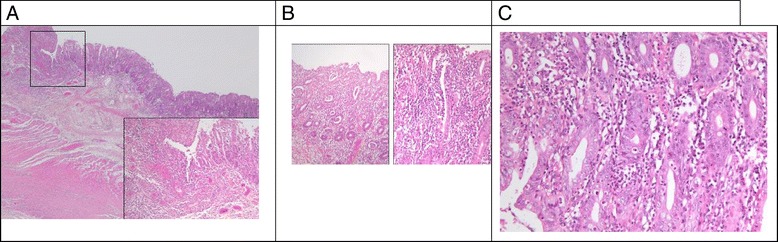
**Colon biopsy of patient UPN5131. (A)** Low magnification of colic resection: mucosal lesion with acute inflammatory infiltrate and ulceration. **(B)** Higher magnification: acute inflammatory infiltrate of the lamina propria with destruction of the epithelium, forming crypt abscesses with numerous neutrophils. **(C)** High magnification showing crypt abscesses: neutrophilic infiltration of the lamina propria with destruction of some glands.

IBD or Crohn’s disease was diagnosed on gut biopsy in three patients. The first symptoms were recurrent abdominal pains and chronic diarrhea. Age at first manifestation of the digestive symptoms was young (between the ages of 6 and 10 years) even if gut biopsy to document IBD was performed later between ages 10 and 15 years. No patients in our survey were treated by anti-TNF alpha therapy, and oral steroids appeared sufficient to improve symptomatology. In two patients, bowel stenosis led to a partial colectomy. Colon biopsy of patient UPN5131 showed acute inflammatory infiltrate of the lamina propria with destruction of the epithelium, forming crypt abscesses with numerous neutrophils, suggesting a local process attracting the neutrophils, which became activated but could not fulfill their roles (Figure 5). Each member of pedigree A had an objective steatorrhea (>3 g/24 h); the cause of this steatorrhea is not clear because no pancreatic insufficiency was diagnosed, but pancreatic enzyme resulted in symptom correction. One other patient also presented with steatorrhea. One patient had a severe gastroesophageal reflux. Of 14 patients, eight had growth failure with weight < -3SD and height < -2SD.

### Central nervous system and neurodevelopment

Seven patients had mental delay or learning difficulties. One of them had an abnormal cerebral MRI with sus-tentorial bilateral brain atrophy. This patient, who died at age 5 years from septicemia, presented with severe motor and psychomotor developmental delay. Delay in psychomotor development in the first years of life with age of walking between 18 and 24 months was observed in half of the cases but did not preclude ultimately typical development.

### Other pathologies

Six of fourteen patients had variable features of facial dysmorphology, namely frontal bossing, thick lips, a broad nasal bridge, and prognathism. One patient had facial features present in Kabuki syndrome. One patient had a portal cavernoma, and one had Raynaud syndrome. Two patients in our cohort had polyarthritis treated with oral steroids, and two patients had bilateral hearing loss. Puberty was delayed in one patient.

### Long-term outcome

**Figure 6:**
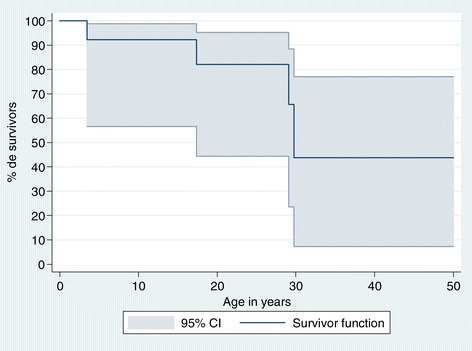
**Kaplan–Meier survival curve with 95% confidence interval (CI) for the 14 patients with** ***G6PC3*** **mutations in France.**

The 20-year survival and 30-year survival rates were 82% and 44%, as shown on Figure 6 Eight patients reached age 18 years at minimum. As adults, two had a limited burden of disease and were able to work, one after achieving a terminal PhD and the other having no diploma. One of these two patients was the mother of a healthy child. Six other adults experienced severe limitations and were not able to perform regular work. In one case (UPN5194), several limitations such as cardiac and respiratory insufficiency, hypogonadism, and hypothyroidism were attributable to the hematopoietic stem cell transplantation performed 20 years earlier. As described, two patients died at around age 30 years, suddenly and for unknown causes. The circumstances of the deaths suggested that acute cardiac dysrhythmia might have been responsible.

## Discussion

We report a national survey of 14 cases of congenital neutropenia related to *G6PC3* mutations. A total of 61 cases have been reported [2,14-28] since the initial description of the gene [2]. Because one of our cases has already been described [2], here we have added 13 original cases to the literature.

Our national survey allowed us to estimate the incidence rate at birth, which is estimated at 0.4/10^6^ births in the French population (95% confidence interval, 0.009–0.9/10^6^). The initial description [2] was based on a population of Aramean ancestry, but we failed to confirm this as a common feature, and this disease appears to be ubiquitous. Its terminology seems to be somewhat controversial because several names have been proposed, including SCN type 4 (MIM 610738) [19] or Dursun syndrome (MIM 613034 or 612541) [26], in addition to *G6PC3* neutropenia. The assignment of the number 4 for SCN seems rather inconsistent with the order of discovery of the gene in congenital neutropenia. Dursun syndrome is not really appropriate because we report two publications of cases, one from 1982 [9] and one from 1994 [8], the latter documented to be in relation to the *G6PC3* mutation. The term ‘congenital neutropenia associated with *G6PC3 mutation*’ appears to be the least controversial and the most accurate.

Our report adds not only a significant number of cases but also several important morbid associations. We describe 14 patients from 8 families, in which six patients are consanguineous. This neutropenia presents a large extended phenotype. Among the 74 published cases, including ours, only four (5%) were diagnosed without any comorbidities [20]. The most frequent comorbidities are prominent skin veins, urogenital malformations, and heart malformations [2,22]. Our survey clarifies and extends the phenotype. With regard to cardiac abnormalities, atrio-septal defect and patent ductus arteriosus are the most frequently associated while primary pulmonary hypertension has been described in 5/57 patients. Two patients in our cohort also had pulmonary hypertension while two had aortic regurgitation. Valvular anomalies have been also described, but it is important to consider potentially lethal paroxysmal heart rhythm trouble as a complication of *G6PC3* mutations. Such complications can be preceded by early detection of Wolff-Parkinson-White abnormalities but also may have a sole acute expression in young adults, leading to sudden death.

Myopathic syndrome was mentioned briefly in the initial case publication (case #9) [2], and we provide here more detailed clinical pathological findings of this case. This myopathic syndrome is not attributable to a permanent myopathic defect but is rather a critical event involving recurrent cramps with effort. This manifestation was not mentioned in the literature and appeared, on a pathological basis, to be related to glycolytic metabolism impairment. In the overall series, neurological development appeared to be mildly delayed in the majority of cases. In one case among 14, the delay was significant and was associated with cerebral atrophy on MRI while in the other cases, it seemed limited and transient, and one patient was able to pursue studies up to the university level.

Our survey demonstrated that *G6PC3* neutropenia may also be associated with myelodysplasia and AML. Our patient presented a myelodysplasia at age 14 years, followed by AML 2 with translocation (8;21) six months later. Such complications are already well known in the great majority of congenital neutropenias [10,29], including glycogen storage type Ib [30,31], but have not been described so far in the 61 cases presented in the literature. The case described here is the first case of a malignancy in this type of neutropenia. This leukemia occurred in the absence of G-CSF exposure and suggests that the risk of leukemia, as in other congenital neutropenias, is associated with and results from the genetic defect itself. Of note, hematopoietic stem cell transplant was a curative option for this patient, who has survived 20 years past this event and is still alive. Today, most patients with *G6PC3* deficiency receive G-CSF, and a high dose of G-CSF is correlated with an increased risk of leukemia; thus, these data suggest the need to closely monitor the possibility of leukemia in this patient group.

The occurrence of IBD in this population appears high. With three cases out of 14 here and five cases documented in the literature [6,22,26], IBD is an important comorbidity of *G6PC3* neutropenia. The initial symptoms in this series were recurrent abdominal pains. Such an association is close to that described in glycogen storage disease type Ib [32]. IBD appeared quite serious, and two of our three patients had a bowel stenosis requiring partial colectomy. IBD was also associated with chronic polyarthritis in one patient. This polyarthritis involved the large joints but also the small joints and was not destructive. In addition to IBD, arthritis can be controlled by oral low dose steroid, which needs to be prolonged. Such an inflammatory process was not associated with autoimmune markers or an associated infection and suggests more an auto inflammatory process.

Our survey offers greater insight into the description of the hematological features of *G6CP3* neutropenia. In the initial description by Botzug et al. [2], early myeloid blockage was observed, but normal myeloid maturation and myelokathexis features have been documented in two cases with *G6PC3* neutropenia [28]. When sequential bone marrow aspiration is done, a bone marrow blockage can occasionally be seen. However, our results, based on a centralized review of all available bone marrow smears in our survey, demonstrated that the most regular aspect of bone marrow is the absence of maturation arrest in this genetic disorder.

As described in one case report, we confirm that in addition to lymphopenia, *G6CP3* neutropenia involves a relative deficiency of naive T cells. These immunological features are not associated with IBD or chronic viral infections and seem likely to be secondary to chronic infections related to the neutropenia.

Thus far among the 61 described cases, four deaths have been reported, one at birth [3], one at 9 months from sepsis [14], and two at 18 months [17]. In the current series, with the largest follow-up reported so far, we observed a higher death rate, mainly in young adults. One death from sepsis occurred at age 5 years, but the three other deaths occurred in young adults, one at age 19 years from the final stage of pulmonary insufficiency and two at around age 30 years, both sudden deaths suggesting acute cardiac dysrythmia.

Our study was not oriented to the pathophysiology of the *G6PC3* defect; however, the very large number of clinical manifestations related to this defect highlights the ubiquitous role of the glucose-6-phosphatase subprotein 3. This protein belongs to the glucose-6-phosphatase complex, which has the major role of releasing glucose from glucose-6-phosphate, a key step in neoglucogenesis. This pathway, however, is not really altered in the human G6PC3 defect, and patients had no impairment in glucose homeostasis. Rather, mutations in this protein affect embryogenesis of the heart, urogenital tract, and central nervous system. In addition, this defect results in a very specific neutropenia characterized by a neutrophil release defect rather than by a maturation arrest and appears to be a consequence of the endoplasmic reticulum stress pathway as well as the glycosylation of proteins. Rigorous mass spectrometric glycomic profiling has shown that most of the complex-type antennae that characterize the neutrophil N-glycome of healthy individuals are severely truncated in patient neutrophils. A comparable truncation of the core 2 antenna of the O-glycans has also been observed. This aberrant neutrophil glycosylation is predicted to have profound effects on neutrophil function and merit designation of this syndrome both syndromes as a new class of congenital disorders of glycosylation [33].

## Abbreviations

ALC: Absolute lymphocyte count
AMC: Absolute monocyte count
ANC: Absolute neutrophil count
GCSF: Granulocyte colony-stimulating factor
G6PC3: Glucose-6-phosphatase, catalytic subunit 3 gene
Hb: Hemoglobin
IUGR: Intrauterine growth retardation
IQR: Interquartile range
SD: Standard deviation
SF: Shortening fraction
UPN: Unique patient number
WBC: White blood cell count
IBD: Inflammatory bowel disease
